# Expression Profiling and Functional Analysis of Candidate Odorant Receptors in *Galeruca daurica*

**DOI:** 10.3390/insects13070563

**Published:** 2022-06-21

**Authors:** Jing-Hang Zhang, Ling Li, Na Li, Yan-Yan Li, Bao-Ping Pang

**Affiliations:** 1 Research Center for Grassland Entomology, Inner Mongolia Agricultural University, Hohhot 010018, China; zhangjinghang0508@163.com (J.-H.Z.); lling@imau.edu.cn (L.L.); liyanyan@imau.edu.cn (Y.-Y.L.); 2 Erdos City Extension Center for Agriculture and Animal Husbandry Technology, Erdos 017200, China; lina445899244@163.com

**Keywords:** electroantennogram, expression profile, RNA interference

## Abstract

**Simple Summary:**

Odorant receptors (ORs) play an important role in the olfactory system in insects. However, there is no functional research on the odorant receptors of *Galeruca daurica*. In this study, 21 OR genes were identified from the transcriptome database of *G. daurica* adults. Most *GdauORs* were mainly expressed in antennae, and the expression levels of *GdauORs* in adults were affected by age. When *GdauOR4*, *GdauOR15*, and *GdauORco* were silenced by RNAi, the electrophysiological responses to host plant volatiles were significantly decreased.

**Abstract:**

*Galeruca daurica* (Joannis) is an oligophagous pest in the grasslands of Inner Mongolia, China, which feed mainly on *Allium* spp. Odorant receptors (ORs) play an important role in the olfactory system in insects, and function together with olfactory co-receptor (ORco). In this study, 21 OR genes were identified from the transcriptome database of *G. daurica* adults, and named *GdauOR1-20* and *GdauORco*. The expression profiles were examined by RT-qPCR and RNA interference (RNAi) and electroantennogram (EAG) experiments were conducted to further identify the olfactory functions of *GdauOR4*, *GdauOR11*, *GdauOR15*, and *GdauORco*. It was found that 15 *GdauORs* (*OR1*, *OR3-6*, *OR8*, *OR11-13*, *OR15*, *OR17-20*, and *ORco*) were mainly expressed in antennae, and the expression levels of *GdauORs* in adults were affected by age. When *GdauOR4*, *GdauOR15*, and *GdauORco* were silenced by RNAi, the electrophysiological responses to host plant volatiles were significantly decreased in *G. daurica*. This study lays a necessary foundation for clarifying the mechanism on finding host plants in *G. daurica*.

## 1. Introduction

A sophisticated olfactory system is a key physiological element for survival and reproduction in insects [1]. In the olfactory system, insects detect semiochemicals through interactions with various olfactory proteins, including odorant-binding proteins (OBPs) and chemosensory proteins (CSPs) which bind odors in lymph fluid, odorant receptors (ORs), and ionotropic receptors (IRs) that convert chemical signals into nerve electrical signals through ion channels, as well as odorant-degrading enzymes (ODEs) that are considered to decompose odorants [1,2]. Olfactory co-receptor (Orco) was initially identified as a member of the OR family in *Drosophila* [3]. During the initial olfaction process, odorant receptors (ORs) expressed on the dendrites of olfactory sensory neurons (OSNs) play a central role in converting the chemical signals into electrical signals via the formation of heteromeric complexes that operate as odorant-gated ion channels with an Orco [4]. Most species of insects express just one Orco and a distinct complement of OR, ranging from just four members in *Calopteryx splendens* to more than 300 in *Tribolium castaneum* [5,6,7]. Numerous studies have shown that insect ORs are mainly expressed in olfactory organs, for example, 60 OR genes in *Microplitis mediator* have high expression in both male and female antennae [8], and 38 ORs in *Aethina tumida* were predominately expressed in antennae [9]. Functional characterizations of odorant receptors have been verified in many insect species so far. The main research methods are CRISPR/Cas9 system, HEK293 cell line, *Drosophila* aT1 or ab3 system, *Xenopus* oocyte model system and RNA interference techniques [10,11,12,13,14,15]. In the Coleoptera, a large number of chemoreceptor genes have been studied [16,17].

*Galeruca daurica* (Joannis) (Coleoptera: Chrysomelidae) had an abrupt outbreak on the Inner Mongolia grasslands in 2009, and since then, the occurring range has been expanding in recent years [18]. This beetle is an oligophagous pest, which feeds mainly on *Allium* spp, and *A. mongolicum* is the optimal host plant [19]. This feeding habit of *G. daurica* implies an important role of olfaction in searching for specific host plants. According to our previous study, six compounds of *A. mongolium* could stimulate the strong EAG response of *G. daurica*, including diallyl sulfide, diallyl disulphide, (Z)-2-hexen-1-ol, 2-hexenal, methyl benzoate, and hexanal [20].

Up to now, some olfactory-related genes have been studied in *G. daurica*. 29 OBPs and 10 CSPs genes were identified and the expression profiles were analyzed by RT-PCR and RT-qPCR [21,22]. Meanwhile, the binding properties of five OBPs (GdauOBP1, GdauOBP6, GdauOBP10, GdauOBP15 and GdauOBP20) and two CSPs (GdauCSP4 and GdauCSP5) were analyzed using a number of ligands of *A. mongolium* in competitive binding assays [23,24]. The olfactory functions of *GdauOBP15* and *GdauCSP5* were explored by RNAi [23]. However, there is no functional research on the odorant receptors of *G. daurica*. In this study, the odorant receptor genes of *G. daurica* were identified from transcriptome data and the expression profiles were analyzed by RT-qPCR. The functional studies of *GdauOR4*, *GdauOR11*, *GdauOR15* and *GdauORco* were carried out by RNA interference (RNAi) combined with electroantennogram. Our study aims to clarify the molecular mechanisms on chemical sensitivity of *G. daurica*, and lay the necessary foundation for finding the target gene to control this insect with green prevention and control technique.

## 2. Materials and Methods

### 2.1. Insects and Samples Collection

The larvae of *G. daurica* were collected from Xilinhot, Inner Mongolia, China (43°54′53″ N, 115°39′13″ E) in May 2021. Insects were reared on *A. mongolium,* and maintained at 26 ± 1 °C under 60–80% humidity and a 16 h light: 8 h dark photoperiod. The tissues (antennae, heads without antennae, thoraxes without wings and legs, abdomens, legs, and wings) were collected from 3-day-old male and female adults (unmated) by using scalpel and forceps in the forenoon. Antennae from different days after eclosion (1-day-old, 3-day-old, 7-day-old, 15-day-old, 25-day-old, 45-day-old, and 90-day-old) of male and female adults were collected every morning. Among them, the 1-day-old, 3-day-old, and 7-day-old adults were in the active stage and foraged heavily. The 15-day-old, 25-day-old, and 45-day-old adults were in the diapause stage and did not forage. The 90-day-old adults were released from diapause, which were in the mating stage and resumed foraging. Each group of samples consisted of 20 individuals, and three biological replicates were set. All samples were frozen in liquid nitrogen as they were collected and stored at −80 °C until RNA extraction.

### 2.2. Identification of OR Transcripts

We identified putative OR genes by searching the transcriptome database of *G. daurica* adults assembled in our laboratory [25]. Putative OR genes were searched using “olfactory receptor” and “odorant receptor” as the key words to screen the annotated sequences in the transcriptome database. All putative OR genes were manually confirmed using the Blastx program against the NR nucleotide database at NCBI with a cut-off E-value 10^−5^. The open reading frames (ORFs) of OR genes were predicted using the ORF Finder (http://www.ncbi.nlm.nih.gov/gorf/gorf.html, accessed on 11 November 2020).

### 2.3. Total RNA Isolation and cDNA Synthesis

Total RNA of each sample was isolated using TaKaRa MiniBEST Universal RNA Extraction Kit (TaKaRa, Dalian, China) following the manufacturer’s technical manual, and the RNA integrity was checked using 1.5% agarose gel electrophoresis. The extracted RNA was quantified in NanoDrop™ 2000 spectrophotometer (Thermo Fisher, Waltham, MA, USA). cDNA was synthesized using the PrimeScript^TM^ 1st Strand cDNA Synthesis Kit (TaKaRa, Dalian, China), and 1 μg RNA was used for cDNA synthesis according to the instructions.

### 2.4. Quantitative Real-Time PCR (RT-qPCR) Measurement

Gene expression profiles were analyzed by RT-qPCR. All primers for RT-qPCR were designed by Primer Premier 5.0 (http://www.premierbiosoft.com/primerdesign/index.html, accessed on 10 December 2020) (Table S1). RT-qPCR were performed using the FTC-3000P Real-Time Quantitative Thermal Cycler (Funglyn Biotech, Markham, ON, Canada) with BRYT Green^®^ dye (GoTaq^®^ qRT-PCR Master Mix, Promega, Madison, WI, USA) as the fluorescence reporter. The succinate dehydrogenase complex gene (*SDHA*) was used as a reference gene [26]. A 10-fold dilution series of antennal cDNA was employed to construct a standard curve to determine the PCR efficiency. All amplification efficiencies in the RT-qPCR analysis ranged from 85.8% to 103%. Experiments were performed in a 10 μL reaction mixture, including 1 μL cDNA (10 ng/μL), 0.2 μL forward primer (10 μmol/L), 0.2 μL reverse primer (10 μmol/L), 5 μL BRYT Green dye, and 3.6 μL RNase free water, and repeated three times for each sample. The RT-qPCR reaction was carried out according to the following procedure: initial denaturation at 95 °C of 10 min, followed by 45 cycles of 95 °C for 15 s, and annealing at 60 °C for 1 min, and analysis of the melting curve at the last. There was a specific single peak in each reaction in the dissociation stage. The relative quantification of each gene expression level was analyzed using the 2^−ΔΔCt^ method [27]. The expression level of *GdauOR1* in male antennae was used as the control in the expression profiles of different tissues. The expression level of each gene in 1-day-old male antennae was used as the control in the expression profiles of different days after eclosion.

### 2.5. RNA Interference of GdauOR4/GdauOR11/GdauOR15/GdauORco

RNA interference (RNAi) primers containing T7 promoter were designed to the coding sequence of *GdauOR4*, *GdauOR11*, *GdauOR15*, and *GdauORco* (Table S2). PCR was performed in a 25 μL reaction (1 μL cDNA, 1 μL forward primer, 1 μL reverse primer, 9.5 μL Premix Taq^TM^, and 12.5 μL RNase free water) with a thermocycler (T100^TM^ Thermal Cycler, Bio-Rad, Hercules, CA, USA), conditions consisting of: 94 °C for 3 min followed by 35 cycles of 94 °C for 30 s, 65 °C for 30 s (each gene used a primer-specific temperature), 72 °C for 1 min, and a final extension at 72 °C for 10 min. Amplification products were purified and connected to the pGEM-T Easy vector, and then transformed into the competent-cell Escherichia coli DH5α (TaKaRa, Dalian, China). Plasmids were sequence verified and used as templates to synthesize double-stranded RNAs (dsRNA) through the kit of T7 RiboMAX^TM^ Express RNAi System (Promega, Madison, WI, USA). The quality of dsRNA was detected by 1.5% agarose gel electrophoresis. The concentration was detected by NanoDrop™ 2000 spectrophotometer. Finally, the dsRNA was diluted to 1000 ng/µL in enzyme-free water and stored at −80 °C until use.

The 3-day-old female and male adults were selected as test insects. Two µL dsRNA was injected into the intersegmental membrane between the fourth and fifth abdominal segments of *G. daurica* using a microinjector (Shimadzu, Kyoto City, Japan). The experiments were divided into 5 treatment groups (ds*OR4*-injected, ds*OR11*-injected, ds*OR15*-injected, ds*ORco*-injected and ds*GFP*-injected) with 3 biological replicates per group and 20 individuals per replicate. The treated insects were reared under the above conditions. Antennae of *GdauORco* group were collected separately for interference efficiency measurement by RT-qPCR at 24, 48, 72, and 96 h after injection. Antennae of *GdauOR4*, *GdauOR11*, and *GdauOR15* groups were collected for interference efficiency measurement at 48 h after injection. The group of ds*GFP*-injected was treated as control.

### 2.6. EAG Recordings

Electroantennogram (EAG) was used to record the antennal responses to 13 volatiles of *A. mongolium* (Table 1) [20]. These compounds were dissolved in mineral oil (Bio-Rad, USA) at 10 μg/μL. The mineral oil was also set up as a blank control. An antenna was cut off from the base, and cut 0.2 mm off the tip, then connected between the electrodes by two silver wires and two capillary glass tubes filled with physiological saline (0.9% sodium chloride injection). The antennal tip and base were inserted into the capillary tubes with 0.5 mm each. Filter paper strips (5 × 15 mm) were loaded with 10 µL of each solution, and transferred to the Pasteur pipette. The top of the pipette was inserted into the small hole in the wall of the tube, which was connected to an air stimulus controller (CS-55; SynTech, Kirchzarten, Germany). The signals were detected by a high-impedance amplifier (IDAC-2; SynTech, Kirchzarten, Germany) and analyzed using SynTech software (GC-EAD 2014 v1.2.5). The stimulation was started with a pulse duration of 0.2 s until the baseline stabilized. The flow rate was 4 mL/s. The EAG responses were recorded during odor stimulation, and six female or male antennae of 3-day-old adults at 48 h after injection were used for each volatile odorant recording.

### 2.7. Data Analysis

All data were statistically analyzed using SPSS Statistics 18.0. In the expression profile analysis, the differences between males and females were compared using *T*-test. Duncan’s test was performed for the differences between different treatments using One-Way ANOVA, and *p* < 0.05 was taken as the level of statistical significance. RNA interference and EAG analysis were analyzed using *T*-test.

## 3. Results

### 3.1. Identification of Putative OR Genes in G. daurica

A total of 21 putative OR genes were identified from the transcriptome database of *G. daurica* adults, which were named as *GdauOR1-20* and *GdauORco*. These sequences data have been submitted to the GenBank Datebase of NCBI under accession numbers MK691770-MK691790 (Table 2).

### 3.2. Expression Profile Analysis of G. daurica OR Genes

**Figure 1:**
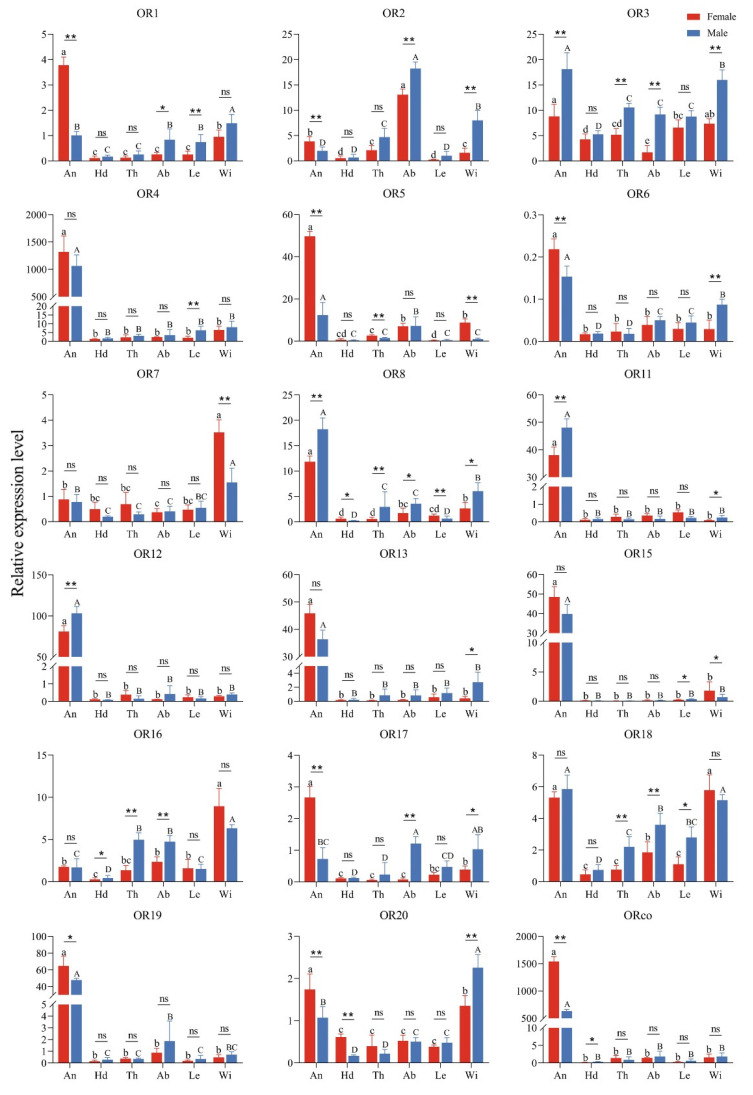
Expression profiles of *GdauORs* in different tissues of female and male adults. An: Antennae; Hd: Head without antennae; Th: Thorax; Ab: Abdomen; Le: Leg; Wi: Wing. Error bars represent the standard error of three independent experiments. Different capital and small letters above bars indicate significant difference among different tissues of females and males, respectively (Duncan’s test; *p* < 0.05). The asterisk above bars indicates significant difference between males and females (*T*-test; *: *p* < 0.05; **: *p* < 0.01; ns: No significant difference).

The expression profiles of *GdauORs* in different tissues (Figure 1) showed that the expression levels of fifteen genes in antennae were significantly higher than those in other tissues, including *GdauOR1*, *GdauOR3*-*6*, *GdauOR8*, *GdauOR11*-*13*, *GdauOR15*, *GdauOR17*-*20*, and *GdauORco*. Among them, the expression levels of seven genes (*GdauOR1*, *GdauOR5*-*6*, *GdauOR17*, *GdauOR19*-*20*, and *GdauORco*) in female antennae were significantly higher than those in male antennae, while four genes (*GdauOR3*, *GdauOR8*, *GdauOR11*, and *GdauOR12*) were opposite. Significantly, the genes with the highest expression levels in antennae were *GdauOR4* and *GdauORco*. In addition, the expressions of *GdauOR3*, *GdauOR7*, *GdauOR16*, *GdauOR18* and *GdauOR20* were significantly enriched in wings. Peculiarly, *GdauOR2* was abundantly expressed in abdomens, and the expression level in males was significantly higher than that in females. While *GdauOR9*, *GdauOR10*, and *GdauOR14* were not shown in the graph, because their expression levels were too low.

**Figure 2:**
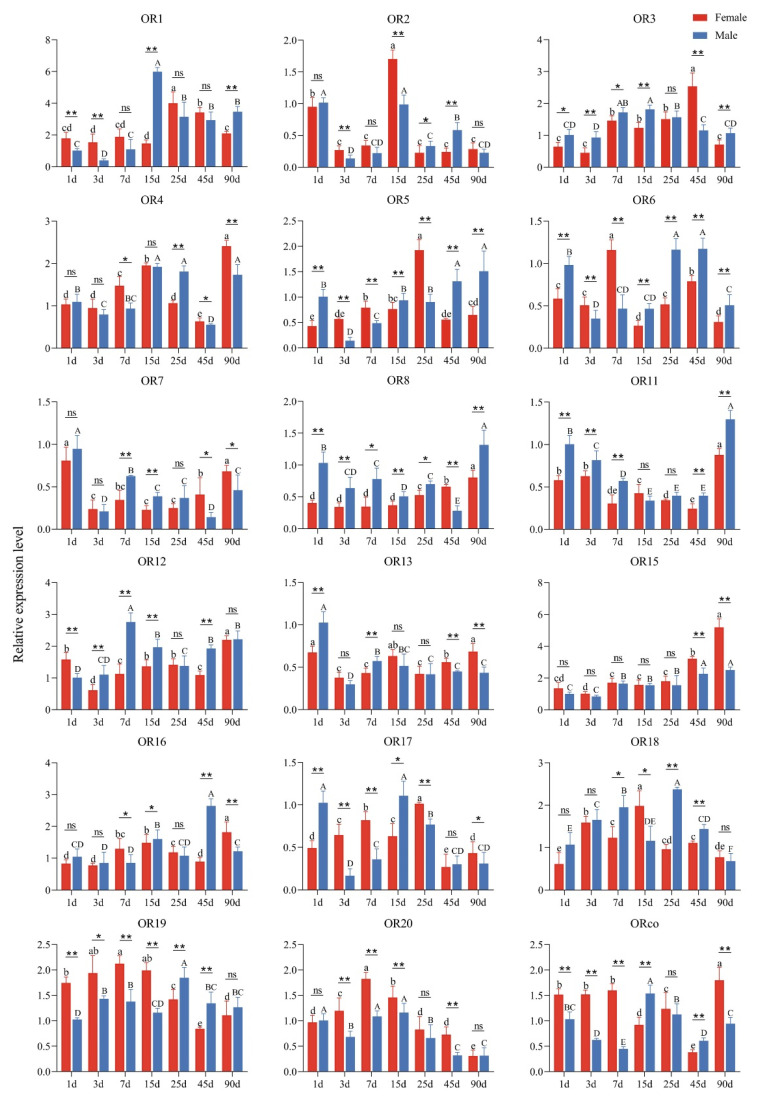
Expression profiles of *GdauORs* at different days after eclosion of antennae in female and male adults. 1 d: 1-day-old; 3 d: 3-day-old; 7 d: 7-day-old; 15 d: 15-day-old; 25 d: 25-day-old; 45 d: 45-day-old; 90 d: 90-day-old. Error bars represent the standard error of three independent experiments. Different capital and small letters above bars indicate significant difference among different developmental days of female and male, respectively (Duncan’s test; *p* < 0.05). The asterisk above bars indicates significant difference between males and females (*T*-test; *: *p* < 0.05; **: *p* < 0.01; ns: No significant difference).

The expression profiling results of *GdauORs* at different days after eclosion (Figure 2) showed that *GdauOR1* had a higher expression level in males at 15 d, while *GdauOR2* had a higher expression level in females at 15 d. *GdauOR3* had a higher expression level in females at 45 d. *GdauOR4* and *GdauORco* had a higher expression level in females at 90 d. *GdauOR5* had a higher expression level in females at 25 d. *GdauOR6* had a higher expression level in females at 7 d and males in 25 d as well as 45 d. *GdauOR7* and *GdauOR13* had a higher expression level in males at 1 d. *GdauOR8* and *GdauOR11* had a higher expression level in males at 90 d. *GdauOR12* had a higher expression level in males at 7 d. The expression level of *GdauOR15* increased with the days after eclosion, and highest in females at 90 d. *GdauOR16* had a higher expression level in males at 45 d. *GdauOR17* had a higher expression level in males at 1 d and 7 d. *GdauOR18* had a higher expression level in males at 25 d. *GdauOR19* had almost the same expression levels for all stages of adults with few exceptions. *GdauOR20* had a higher expression level in females at 7 d. Each gene showed significant differences between male and female antennae at different days of eclosion. It is noteworthy that there were significant differences in expression levels of 13 genes between male and female antennae at 90 d, when they were in the mating stage. Among them, the expression levels of *GdauOR4*, *GdauOR7*, *GdauOR13*, *GdauOR15*, *GdauOR16*, *GdauOR17*, and *GdauORco* were significantly higher in females than in males, whereas *GdauOR1*, *GdauOR3*, *GdauOR5*, *GdauOR6*, *GdauOR8*, and *GdauOR11* were opposite.

### 3.3. RNA Interference of GdauOR4/GdauOR11/GdauOR15/GdauORco

**Figure 3:**
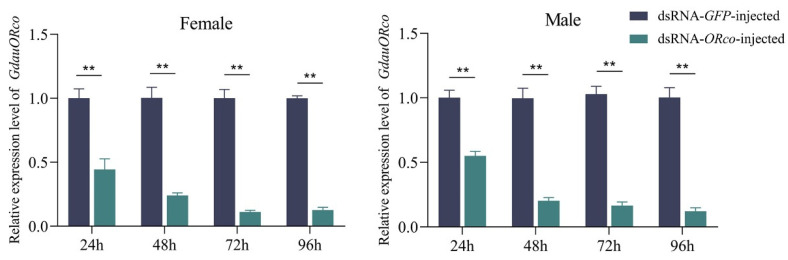
Expression level of *GdauORco* after RNAi. The relative expression levels of *GdauORco* in antennae of dsRNA-injected female and male at different times (*T*-test; **: *p* < 0.01).

**Figure 4:**
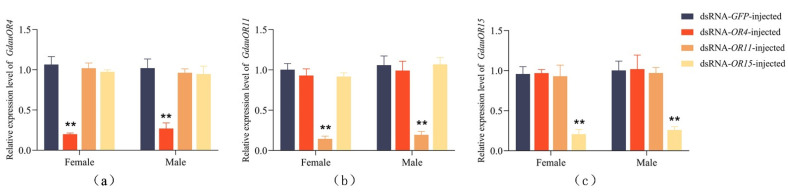
Expression level of *GdauOR4* (**a**), *GdauOR11* (**b**) and *GdauOR15* (**c**) after RNAi 48 h. Bars labeled with different letters are significantly different (*T*-test; **: *p* < 0.01). Columns indicate the mean ± standard error of three independent experiments.

To examine the RNAi efficiency, RT-qPCR was performed. Briefly, injection of dsRNA significantly decreased expression levels of target genes. Compared with the ds*GFP*-injected, when ds*ORco* were injected, the mRNA levels of *GdauORco* in female antennae were decreased by 56%, 76%, 89%, and 87% at 24, 48, 72, and 96 h post-injection, respectively (Figure 3). The mRNA levels of *GdauORco* in male antennae were decreased by 45%, 80%, 83%, and 88% at 24, 48, 72, and 96 h post-injection, respectively. This result indicated that a reduction of RNAi efficiency to about 25% at 48 h post-injection is well processed for *GdauORs*. Similarly, the expression levels of ds*OR4*-injected, ds*OR11*-injected, and ds*OR15*-injected at 48 h post-injection in both female and male antennae were reduced to less than 27% compared with control groups (Figure 4). Among them, RNAi reduced the expression levels of *GdauOR4* to 19.9% and 27% in females and males, *GdauOR11* was reduced to 14.5% and 19.5% in females and males, and *GdauOR15* was reduced to 20.6% and 25.8% in females and males, respectively. There was no significant influence on expression levels of *GdauOR11* and *GdauOR15* when ds*OR4* was injected (Figure 4a), and similarly, injection of ds*OR11* did not affect the expression of *GdauOR4* and *GdauOR15* (Figure 4b); injection of ds*OR15* also did not affect the expression of *GdauOR4* and *GdauOR11* (Figure 4c).

### 3.4. Electroantennogram Analysis

**Figure 5:**
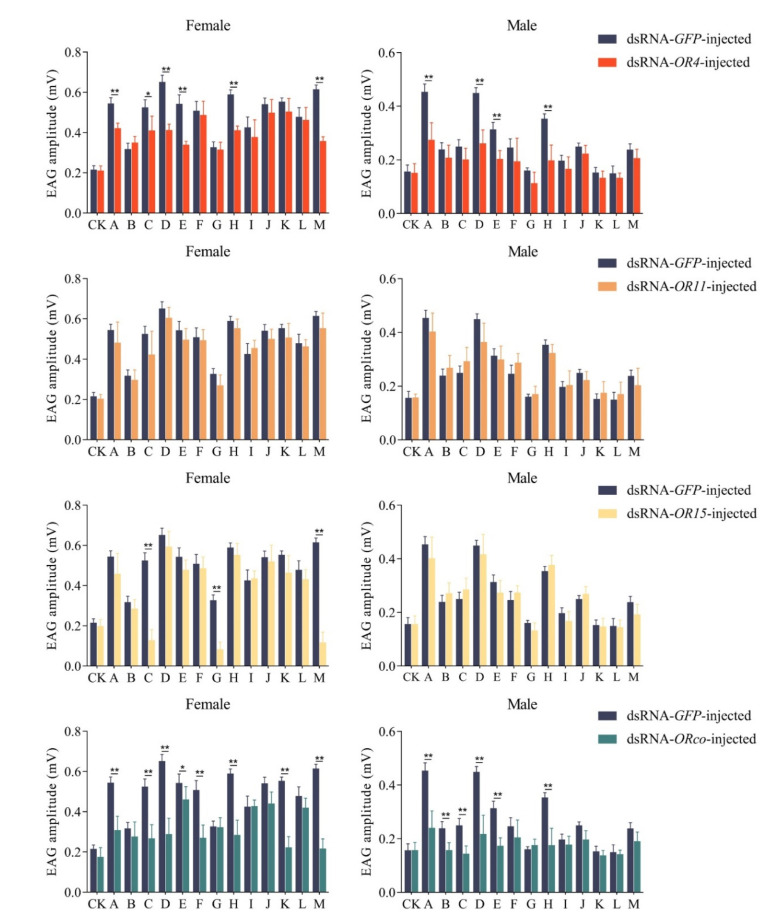
EAG responses of dsRNA-*OR4*-injected, dsRNA-*OR11*-injected, dsRNA-*OR15*-injected and dsRNA-*ORco*-injected in *G. daurica* female and males to various compounds. Columns indicate the mean ± standard error of six independent experiments. (*T*-test; *:*p* < 0.05; **: *p* < 0.01). CK: Mineral oil; A: Diallyl sulfide; B: 1,3-Dithiane; C: Dimethyl trisulfide; D: Diallyl disulphide; E: Diallyl trisulfide; F: 2-Hexen-1-ol; G: Myrcene; H: 2-Hexenal; I: Methyl benzoate; J: Hexanal; K: 1,3,5-Cycloheptatriene; L: p-Xylene; M: Disulfide methyl 2-propenyl.

In order to elucidate the physiological function of *GdauOR4*, *GdauOR11*, *GdauOR15* and *GdauORco* in the perception of host plant volatiles, the electrophysiological responses after RNAi were detected by EAG experiments (Figure 5). Compared with the control group, EAG activity of females in response to six volatiles was significantly reduced when ds*OR4* was injected, including diallyl sulfide, diallyl disulphide, diallyl trisulfide, 2-hexenal, disulfide methyl 2-propenyl (*p* < 0.01), and dimethyl trisulfide (*p* < 0.05). Males showed significantly lower EAG responses to four odors when ds*OR4* was injected, including diallyl sulfide, diallyl disulphide, diallyl trisulfide, and 2-hexenal (*p* < 0.01). In the *dsOR11*-injected group, both females and males had no significant influence on the electrophysiological responses to all tested odors. In ds*OR15*-injected group, EAG activity of females in response to three volatiles was significantly reduced, including dimethyl trisulfide, myrcene, and disulfide methyl 2-propenyl (*p* < 0.01). However, there was no significant influence on the electrophysiological responses when ds*OR15* was injected into males. When *ORco* was silenced, the electrophysiological responses to eight volatiles were significantly decreased in females, including diallyl sulfide, dimethyl trisulfide, diallyl disulphide, 2-hexen-1-ol, 2-hexenal, 1,3,5-cycloheptatriene, disulfide methyl 2-propenyl (*p* < 0.01), and diallyl trisulfide (*p* < 0.05). Meanwhile, the EAG responses of males to six volatiles were reduced significantly, including diallyl sulfide, 1,3-dithiane, dimethyl trisulfide, diallyl disulphide, dimethyl trisulfide, and 2-hexenal (*p* < 0.01).

## 4. Discussion

In this study, we identified 21 OR genes from the adult *G. daurica* transcriptome, less than identified for most other Coleoptera [28,29,30]. We speculate that our use of a high-throughput sequencing approach using whole insects rather than antennae may result in a significant number of olfactory genes being buried. Expression profiling of chemosensory genes are of primary importance for exploring the function and the olfactory recognition mechanism of insects [31,32,33]. The tissue expression profiles of 21 ORs showed that most OR genes (*GdauOR1*, *GdauOR3*-*6*, *GdauOR8*, *GdauOR11*-*13*, *GdauOR15*, *GdauOR17*-*20*, and *GdauORco*) were highly expressed in antennae, which were similar to the results in other species [9]. This result suggested that these ORs might be involved in the chemosensory process [34,35,36]. Notably, *GdauORco* and *GdauOR4* were extremely highly expressed in antennae, suggesting that they may play an important role in the olfactory system. While, *GdauOR9*, *GdauOR10*, and *GdauOR14* had extremely low expression, presumably expressed in the other developmental stages [37,38]. In addition, the expressions of *GdauOR3*, *GdauOR7*, *GdauOR16*, *GdauOR18*, and *GdauOR20* were significantly enriched in wings, and *GdauOR2* were abundantly expressed in abdomens, which suggested that they might be involved in other physiological functions of *G. daurica.* Different days after eclosion expression analysis indicated that the expression levels of *OR*s in antennae were affected by age. The similar results were observed in other olfactory proteins [39]. The expression profile analysis lays a necessary foundation for revealing the olfactory recognition mechanism of *G. daurica*. Four *OR*s, *GdauOR4*, *GdauOR11*, *GdauOR15*, and *GdauORco* were selected for further research in odor detection, because they were highly expressed in antennae.

In a previous study, RNAi technology was effectively used in *G. daurica* by dsRNA injection [23]. Thus, RNAi injection experiments against four ORs were conducted. Same as the previous study, the expression levels were significantly decreased 48 h after dsRNA injection, indicating that dsRNA injection was suitable for target gene interference in *G. daurica*. Furthermore, it has been reported that dsRNA injection can lead to off-target, which means that the expression levels of other non-target genes may be reduced by RNAi [40]. As far as our current work is concerned, there were no significant influence on expression levels of *GdauOR11* and *GdauOR15* when ds*OR4* was injected, and similarly, injection of ds*OR11* did not affected the expression of *GdauOR4* and *GdauOR15,* injection of ds*OR15* also did not affected the expression of *GdauOR4* and *GdauOR11.* This RT-qPCR result is consistent with previous study that the mRNA levels of *HarmPBP1*, *HarmPBP2* and *HarmPBP3* were not affected by each specific dsRNA injection in *Helicoverpa armigera* [41].

Many studies have shown that the involvement of genes in olfactory functions can be ultimately impaired by silencing individual OR genes to influence odor preference [35,42]. The silencing of a single OR gene by RNAi resulted in different electrophysiological changes of *G. daurica* to host plant volatiles. The electrophysiological response of females to six volatiles was significantly reduced when ds*OR4* was injected, including diallyl sulfide, diallyl disulphide, diallyl trisulfide, 2-hexenal, disulfide methyl 2-propenyl, and dimethyl trisulfide. Males showed significantly lower EAG responses to four odors when ds*OR4* were injected, including diallyl sulfide, diallyl disulphide, diallyl trisulfide, and 2-hexenal. Among them, diallyl sulfide, diallyl disulphide, diallyl trisulfide, disulfide methyl 2-propenyl, and dimethyl trisulfide are sulfocompounds with a strong pungent odor, which are the symbolic components of *Allium* plants in Liliaceae such as onion and garlic [43,44,45]. In addition, diallyl disulphide and disulfide methyl 2-propenyl account for 43.16% of the total volatile of *A. mongolicum* [20]. Combined with the expression profile analysis, the expression level of *GdauOR4* was the highest among all *OR*s. Thus, we speculate that *GdauOR4* may be the pivotal receptor for host location of *G. daurica.* For instance, *GmolOR9* was highly expressed in antennae, and silencing *GmolOR9* resulted in reduced sensitivity of *Grapholita molesta* to host volatiles [46].

There was no significant influence on the electrophysiological responses of both females and male to host volatiles after silencing *GdauOR11.* It implied that it may be involved in the recognition of other semiochemicals. In addition, the expression level of *GdauOR11* in male antennae was significantly higher than in females during the whole developmental period of adults. Based on these findings, we speculate that *GdauOR11* may be involved in recognizing pheromones released by females. In *Mythimna separata*, *MsepOR3* was specifically and abundantly expressed in male antennae, and the *Xenopus* oocytes expressing MsepOR3/ORco evinced dose dependent responses to the sex pheromone Z11-16: Ald [47].

EAG activity of females in response to three host plant volatiles (dimethyl trisulfide, myrcene, and disulfide methyl 2-propenyl) was significantly reduced after silencing *GdauOR15*. However, there was no significant influence on the electrophysiological responses in males. Expression profile analysis showed that *GdauOR15* were mainly expressed in antennae. Moreover, the expression level increased gradually with the development of adults, and it was significantly higher in females than in females at 45-days-old and 90-days-old. This implies that *GdauOR15* may have different functions in odor perception between males and females in *G. daurica*, and it may play more important roles in females searching for host plants or suitable oviposition sites. For instance, *HparOR27* was expressed mainly in female antennae, and it was broadly responsive to three host plant volatiles in *Holotrichia parallela* [48].

When *GdauORco* was silenced, the electrophysiological responses to eight host plant volatiles were significantly decreased in females. Meanwhile, the EAG responses of males to six volatiles were reduced significantly. These results suggest that *GdauORco* is necessary for odorant responses of *G. daurica*. ORs cannot assemble, transport, or function in the absence of ORco and the loss of this single receptor results in dramatically impaired olfactory behavior [4]. For instance, the HEK293/MdesOR115 cell line which was devoid of ORco did not respond to compounds in Hessian fly pheromone reception [11]. RNAi reduced the expression of *SaveOrco* to 34.11% in aphids, resulting in weaker EAG responses to plant volatiles and aphid alarm pheromone [49]. Taken together, interference with *GdauORco* resulted in EAG response decreased to eight volatiles in females and six volatiles in males, respectively, these substances include those causing a reduction in EAG response after silencing other three *GdauOR* genes, except myrcene. It indicates that *GdauORco* functionally synergizes with *GdauOR4*, *GdauOR11*, and *GdauOR15* in the olfactory recognition process of *G. daurica*. The response to myrcene may be due to incomplete RNAi or to other unidentified targets OR [50,51].

Although the silencing of a single OR gene by RNAi resulted in electrophysiological changes of *G. daurica* to host plant volatiles, whether it has an impact on behavior needs to be further verified in future experiments.

## 5. Conclusions

Twenty-one OR genes were identified from the adult *G. daurica* transcriptome. Most *OR*s were abundantly expressed in antennae and the expression levels of *OR*s in adults were affected by age. Based on RNAi and EAG experiments, we speculate that *GdauOR4* may be a pivotal receptor for host location in *G. daurica. GdauOR15* may play more important roles in females, but this needs further study for confirmation.

## Figures and Tables

**Table 1:** Odorant stimulus for EAG.

Number	Compound Name	Molecular Formula	Structural Formula	CAS Number	Purity (%)	Manufacturers
A	Diallyl sulfide	C_6_H_10_S	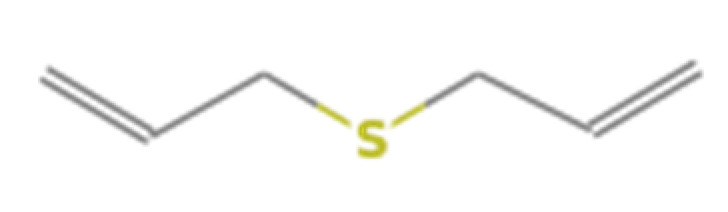	592-88-1	97	Sigma, St. Louis, MO, USA
B	1,3-Dithiane	C_4_H_8_S_2_	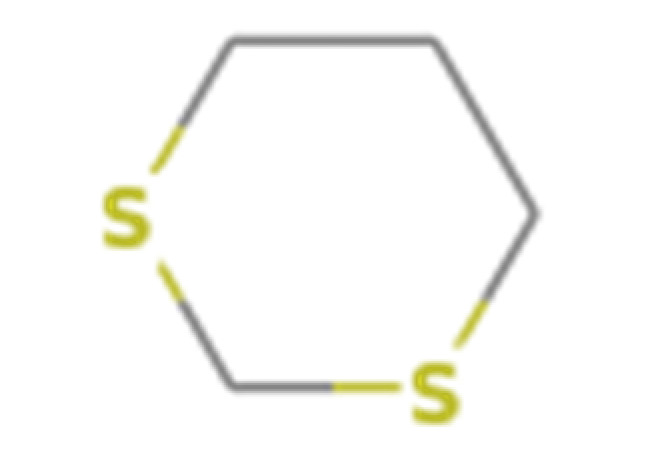	505-23-7	97	Sigma, St. Louis, MO, USA
C	Dimethyl trisulfide	C_2_H_6_S_3_	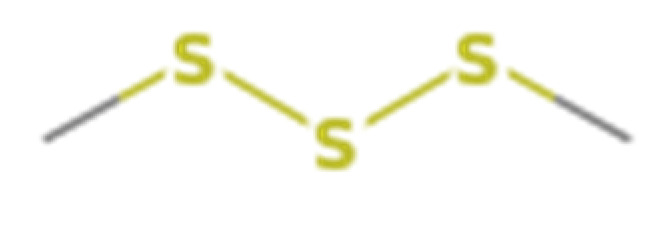	3658-80-8	98	Sigma, St. Louis, MO, USA
D	Diallyl disulphide	C_6_H_10_S_2_	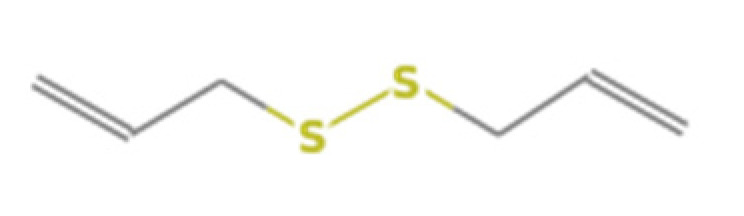	2179-57-9	98	Sigma, St. Louis, MO, USA
E	Diallyl trisulfide	C_6_H_10_S_3_	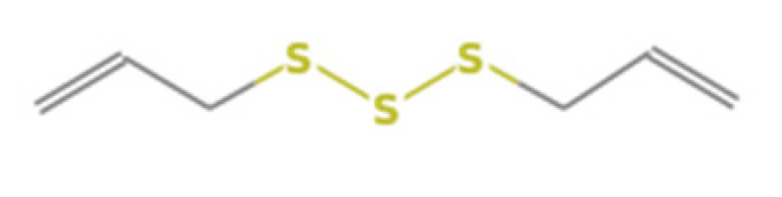	2050-87-5	98	Sigma, St. Louis, MO, USA
F	2-Hexen-1-ol	C_6_H_12_O	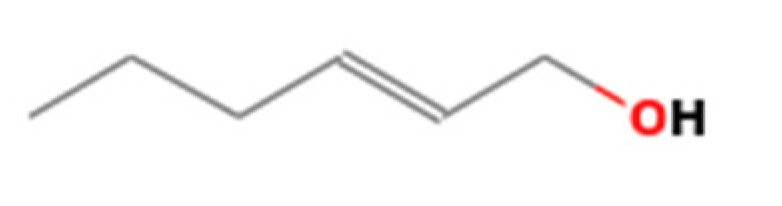	928-95-0	96	Sigma, St. Louis, MO, USA
G	Myrcene	C_10_H_16_	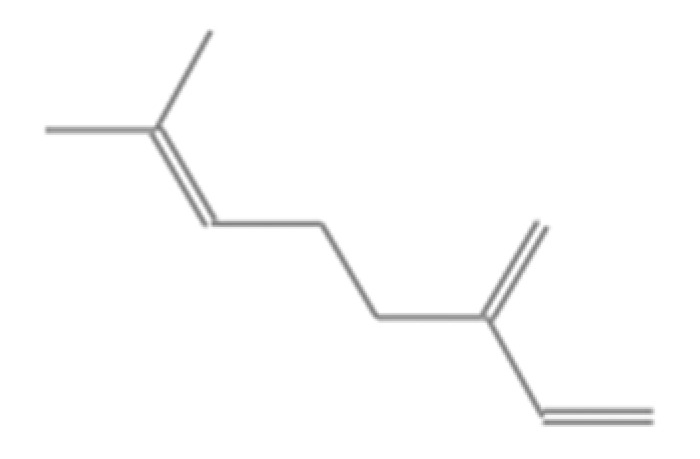	123-35-3	95	Sigma, St. Louis, MO, USA
H	2-Hexenal	C_6_H_10_O	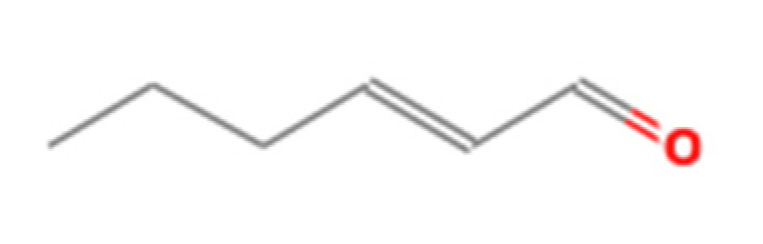	6728-26-3	97	Sigma, St. Louis, MO, USA
I	Methyl benzoate	C_8_H_8_O_2_	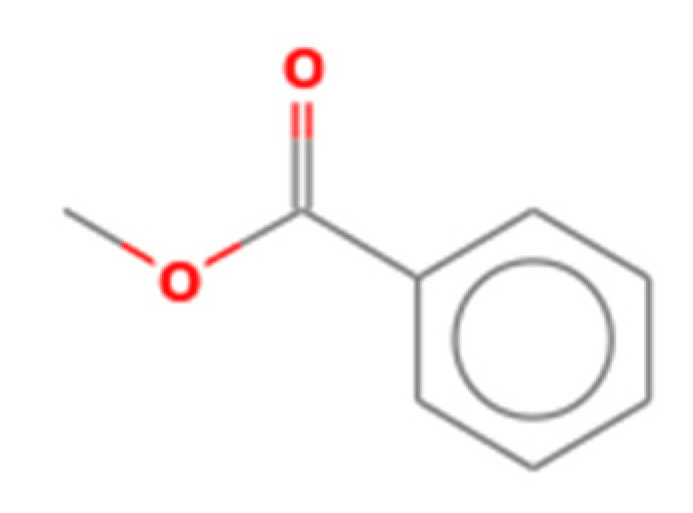	93-58-3	96	Sigma, St. Louis, MO, USA
J	Hexanal	C_6_H_12_O	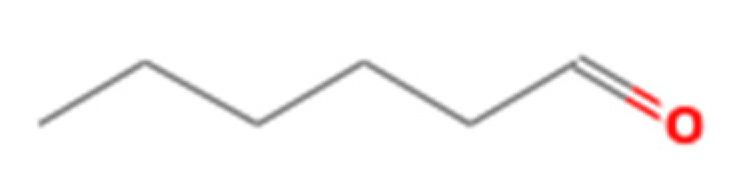	66-25-1	98	Sigma, St. Louis, MO, USA
K	1,3,5-Cycloheptatriene	C_7_H_8_	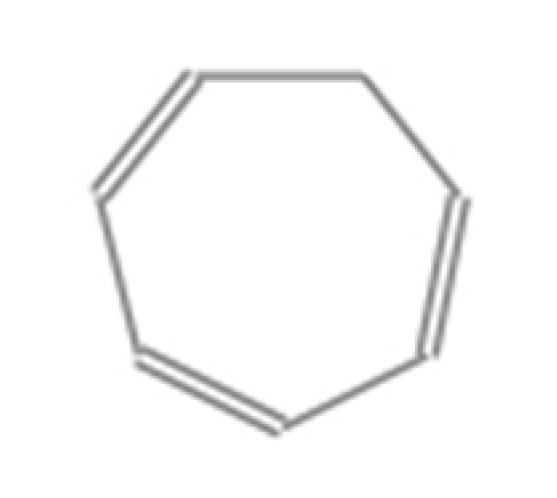	544-25-2	95	Sigma, St. Louis, MO, USA
L	p-Xylene	C_8_H_10_	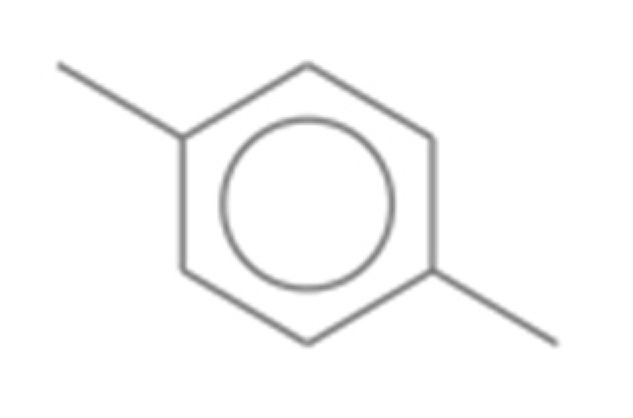	106-42-3	99	Sigma, St. Louis, MO, USA
M	Disulfide methyl 2-propenyl	C_4_H_8_S_2_	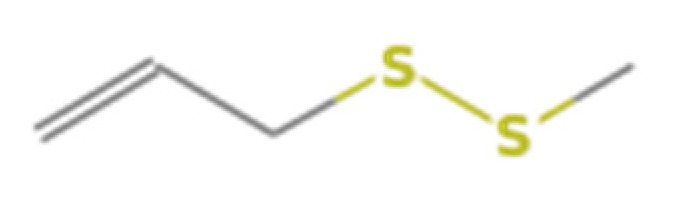	2179-58-0	>90 GC	TCI, Shanghai, China

**Table 2:** List of OR genes in *G. daurica* transcriptome.

Gene Name	Accession Number	ORF (bp)	BLAST Annotation	Query Cover	*E* -Value	Ident (%)	Accession
*GdauOR1*	MK691770	780	odorant receptor 2 (*Pyrrhalta maculicollis*)	98	3 × 10^−53^	38	APC94225.1
*GdauOR2*	MK691771	687	odorant receptor 2 (*Pyrrhalta aenescens*)	98	5 × 10^−107^	68	APC94306.1
*GdauOR3*	MK691772	438	odorant receptor 2 (*Pyrrhalta aenescens*)	93	4 × 10^−34^	47	APC94306.1
*GdauOR4*	MK691773	360	odorant receptor 22 (*Pyrrhalta maculicollis*)	100	4 × 10^−63^	81	APC94232.1
*GdauOR5*	MK691774	351	odorant receptor 83a-like (*Anoplophora glabripennis*)	100	7 × 10^−08^	33	XP_023310752.1
*GdauOR6*	MK691775	318	odorant receptor 25 (*Pyrrhalta aenescens*)	92	1 × 10^−30^	54	APC94326.1
*GdauOR7*	MK691776	285	odorant receptor 21 (*Pyrrhalta maculicollis*)	85	9 × 10^−24^	56	APC94243.1
*GdauOR8*	MK691777	276	odorant receptor 2 (*Pyrrhalta aenescens*)	91	1 × 10^−14^	45	APC94306.1
*GdauOR9*	MK691778	270	dorant receptor 25 (*Pyrrhalta aenescens*)	95	1 × 10^−25^	59	APC94326.1
*GdauOR10*	MK691779	267	odorant receptor 22 (*Pyrrhalta maculicollis*)	100	9 × 10^−44^	76	APC94232.1
*GdauOR11*	MK691780	255	odorant receptor 5 (*Pyrrhalta maculicollis*)	96	3 × 10^−43^	86	APC94229.1
*GdauOR12*	MK691781	246	odorant receptor 5 (*Pyrrhalta maculicollis*)	97	2 × 10^−37^	82	APC94229.1
*GdauOR13*	MK691782	240	odorant receptor 12 (*Pyrrhalta aenescens*)	88	2 × 10^−28^	77	APC94320.1
*GdauOR14*	MK691783	237	odorant receptor Or2-like (*Leptinotarsa decemlineata*)	85	5 × 10^−19^	57	XP_023024059.1
*GdauOR15*	MK691784	234	odorant receptor 3, partial (*Pyrrhalta aenescens*)	100	1 × 10^−26^	65	APC94308.1
*GdauOR16*	MK691785	231	odorant receptor 23, partial (*Pyrrhalta aenescens*)	100	1 × 10^−25^	68	APC94324.1
*GdauOR17*	MK691786	225	odorant receptor 25 (*Pyrrhalta aenescens*)	100	4 × 10^−23^	57	APC94326.1
*GdauOR18*	MK691787	216	odorant receptor (*Anoplophora chinensis*)	97	2 × 10^−11^	43	AUF73043.1
*GdauOR19*	MK691788	213	odorant receptor OR38 (*Colaphellus bowringi*)	94	2 × 10^−18^	54	ALR72581.1
*GdauOR20*	MK691789	168	odorant receptor 25 (*Pyrrhalta aenescens*)	100	8 × 10^−12^	56	APC94326.1
*GdauORco*	MK691790	465	odorant receptor coreceptor, partial (*Agrilus planipennis*)	100	1 × 10^−103^	95	XP_025831003.1

## Data Availability

The data presented in this study are available within the article and Supplementary Materials.

## Supplementary Materials

The following supporting information can be downloaded at: https://www.mdpi.com/article/10.3390/insects13070563/s1, Table S1: List of primers used for qRT-PCR; Table S2: List of primers used in the dsRNA synthesis.

## References

[1] Leal W.S. (2013). Odorant reception in insects: Roles of receptors, binding proteins, and degrading enzymes. Annu. Rev. Entomol..

[2] Pelosi P., Zhou J.J., Ban L.P., Calvello M. (2006). Soluble proteins in insect chemical communication. Cell. Mol. Life Sci..

[3] Vosshall L.B., Wong A.M., Axel R. (2000). An olfactory sensory map in the fly brain. Cell.

[4] Butterwick J.A., Del Marmol J., Kim K.H., Kahlson M.A., Rogow J.A., Walz T., Ruta V. (2018). Cryo-EM structure of the insect olfactory receptor Orco. Nature.

[5] Missbach C., Dweck H.K., Vogel H., Vilcinskas A., Stensmyr M.C., Hansson B.S., Grosse-Wilde E. (2014). Evolution of insect olfactory receptors. eLife.

[6] Ioannidis P., Simao F.A., Waterhouse R.M., Manni M., Seppey M., Robertson H.M., Misof B., Niehuis O., Zdobnov E.M. (2017). Genomic Features of the Damselfly *Calopteryx splendens* Representing a Sister Clade to Most Insect Orders. Genome Biol. Evol..

[7] Engsontia P., Sanderson A.P., Cobb M., Walden K.K., Robertson H.M., Brown S. (2008). The red flour beetle’s large nose: An expanded odorant receptor gene family in *Tribolium castaneum*. Insect Biochem. Mol. Biol..

[8] Wang S.N., Peng Y., Lu Z.Y., Dhiloo K.H., Gu S.H., Li R.J., Zhou J.J., Zhang Y.J., Guo Y.Y. (2015). Identification and Expression Analysis of Putative Chemosensory Receptor Genes in Microplitis mediator by Antennal Transcriptome Screening. Int. J. Biol. Sci..

[9] Wu L., Zhai X., Li L., Li Q., Liu F., Zhao H. (2021). Identification and Expression Profile of Chemosensory Genes in the Small Hive Beetle *Aethina tumida*. Insects.

[10] Li Y., Zhang J., Chen D., Yang P., Jiang F., Wang X., Kang L. (2016). CRISPR/Cas9 in locusts: Successful establishment of an olfactory deficiency line by targeting the mutagenesis of an odorant receptor co-receptor (Orco). Insect Biochem. Mol. Biol..

[11] Andersson M.N., Corcoran J.A., Zhang D.D., Hillbur Y., Newcomb R.D., Lofstedt C. (2016). A Sex Pheromone Receptor in the Hessian Fly *Mayetiola destructor* (Diptera, Cecidomyiidae). Front. Cell. Neurosci..

[12] You Y., Smith D.P., Lv M., Zhang L. (2016). A broadly tuned odorant receptor in neurons of trichoid sensilla in locust, *Locusta migratoria*. Insect Biochem. Mol. Biol..

[13] de Fouchier A., Walker W.B., Montagne N., Steiner C., Binyameen M., Schlyter F., Chertemps T., Maria A., Francois M.C., Monsempes C. (2017). Functional evolution of Lepidoptera olfactory receptors revealed by deorphanization of a moth repertoire. Nat. Commun..

[14] Li R.T., Huang L.Q., Dong J.F., Wang C.Z. (2020). A moth odorant receptor highly expressed in the ovipositor is involved in detecting host-plant volatiles. eLife.

[15] An X., Khashaveh A., Liu D., Xiao Y., Wang Q., Wang S., Geng T., Gu S., Zhang Y. (2020). Functional characterization of one sex pheromone receptor (AlucOR4) in *Apolygus lucorum* (Meyer-Dur). J. Insect Physiol..

[16] Mitchell R.F., Andersson M.N., Blomquist G.J., Vogt R.G. (2021). Olfactory genomics of the Coleoptera. Insect Pheromone Biochemistry and Molecular Biology.

[17] Mitchell R.F., Schneider T.M., Schwartz A.M., Andersson M.N., McKenna D.D. (2020). The diversity and evolution of odorant receptors in beetles (Coleoptera). Insect Mol. Biol..

[18] Zhou X.R., Shan Y.M., Tan Y., Zhang Z.R., Pang B.P. (2019). Comparative Analysis of Transcriptome Responses to Cold Stress in *Galeruca daurica* (Coleoptera: Chrysomelidae). J. Insect Sci..

[19] Hao X., Zhou X.R., Pang B.P., Zhang Z.R., Ma C.Y. (2014). Effects of host plants on feeding amount, growth and development of *Galeruca daurica* (Joannis) larvae (Coleoptera: Chrysomelidae). Sci. Agric. Sin..

[20] Li L., Li N., Pang B.P. (2022). Ultrastructure of antennal sensilla and electroantennographic responses to *Allium mongolium* volatiles in adult *Galeruca daurica* (Coleoptera: Chrysomelidae). Acta Entomol. Sin..

[21] Li L., Zhou Y.T., Tan Y., Zhou X.R., Pang B.P. (2017). Identification of odorant-binding protein genes in *Galeruca daurica* (Coleoptera: Chrysomelidae) and analysis of their expression profiles. Bull. Entomol. Res..

[22] Li L., Zhou Y.T., Tan Y., Zhou X.R., Pang B.P. (2018). Identification and expression profiling of chemosensory protein genes in *Galeruca daurica* (Coleoptera: Chrysomelidae). Acta Entomol. Sin..

[23] Li L., Zhang W.B., Shan Y.M., Zhang Z.R., Pang B.P. (2021). Functional Characterization of Olfactory Proteins Involved in Chemoreception of *Galeruca daurica*. Front. Physiol..

[24] Li L., Tan Y., Zhou X.R., Pang B.P. (2019). Molecular Cloning, Prokaryotic Expression and Binding Characterization of Odorant Binding Protein GdauOBP20 in *Galeruca daurica*. Sci. Agric. Sin..

[25] Li Y.Y., Chen L., Li L., Tan Y., Pang B.P. (2021). Analysis of the transcriptomes of *Galeruca daurica* (Coleoptera: Chrysomelidae) adults at different summer diapause stages. Acta Entomol. Sin..

[26] Tan Y., Zhou X.R., Pang B.P. (2017). Reference gene selection and evaluation for expression analysis using qRT-PCR in *Galeruca daurica* (Joannis). Bull. Entomol. Res..

[27] Livak K.J., Schmittgen T.D. (2001). Analysis of relative gene expression data using real-time quantitative PCR and the 2(-Delta Delta C(T)) Method. Methods.

[28] Li X.M., Zhu X.Y., Wang Z.Q., Wang Y., He P., Chen G., Sun L., Deng D.G., Zhang Y.N. (2015). Candidate chemosensory genes identified in *Colaphellus bowringi* by antennal transcriptome analysis. BMC Genom..

[29] Gonzalez F., Johny J., Walker W.B., Guan Q., Mfarrej S., Jakse J., Montagne N., Jacquin-Joly E., Alqarni A.S., Al-Saleh M.A. (2021). Antennal transcriptome sequencing and identification of candidate chemoreceptor proteins from an invasive pest, the American palm weevil, *Rhynchophorus palmarum*. Sci. Rep..

[30] Wang J., Hu P., Gao P., Tao J., Luo Y. (2017). Antennal transcriptome analysis and expression profiles of olfactory genes in *Anoplophora chinensis*. Sci. Rep..

[31] Wu Z., Ye J., Qian J., Purba E.R., Zhang Q., Zhang L., Mang D. (2022). Identification and Expression Profile of Chemosensory Receptor Genes in *Aromia bungii* (Faldermann) Antennal Transcriptome. Insects.

[32] Scieuzo C., Nardiello M., Farina D., Scala A., Cammack J.A., Tomberlin J.K., Vogel H., Salvia R., Persaud K., Falabella P. (2021). *Hermetia illucens* (L.) (Diptera: Stratiomyidae) Odorant Binding Proteins and Their Interactions with Selected Volatile Organic Compounds: An In Silico Approach. Insects.

[33] Rondoni G., Roman A., Meslin C., Montagné N., Conti E., Jacquin-Joly E. (2021). Antennal Transcriptome Analysis and Identification of Candidate Chemosensory Genes of the Harlequin Ladybird Beetle, *Harmonia axyridis* (Pallas) (Coleoptera: Coccinellidae). Insects.

[34] Zhang R.B., Liu Y., Yan S.C., Wang G.R. (2019). Identification and functional characterization of an odorant receptor in pea aphid, *Acyrthosiphon pisum*. Insect Sci..

[35] Antony B., Johny J., Montagne N., Jacquin-Joly E., Capoduro R., Cali K., Persaud K., Al-Saleh M.A., Pain A. (2021). Pheromone receptor of the globally invasive quarantine pest of the palm tree, the red palm weevil (*Rhynchophorus ferrugineus*). Mol. Ecol..

[36] Ji T., Xu Z., Jia Q., Wang G., Hou Y. (2021). Non-palm Plant Volatile alpha-Pinene Is Detected by Antenna-Biased Expressed Odorant Receptor 6 in the *Rhynchophorus ferrugineus* (Olivier) (Coleoptera: Curculionidae). Front. Physiol..

[37] Di C., Ning C., Huang L.Q., Wang C.Z. (2017). Design of larval chemical attractants based on odorant response spectra of odorant receptors in the cotton bollworm. Insect Biochem. Mol. Biol..

[38] Miao C.L., Hou W., Dong S.L. (2021). Developmental stage-, tissue- and sex-specific expression of three major chemoreceptor gene families in diamondback moth *Plutella xylostella*. J. Plant Prot..

[39] Ali S., Ahmed M.Z., Li N., Ali S.A.I., Wang M.Q. (2019). Functional characteristics of chemosensory proteins in the sawyer beetle *Monochamus alternatus* Hope. Bull. Entomol. Res..

[40] Jackson A.L., Bartz S.R., Schelter J., Kobayashi S.V., Burchard J., Mao M., Li B., Cavet G., Linsley P.S. (2003). Expression profiling reveals off-target gene regulation by RNAi. Nat. Biotechnol..

[41] Dong K., Sun L., Liu J.T., Gu S.H., Zhou J.J., Yang R.N., Dhiloo K.H., Gao X.W., Guo Y.Y., Zhang Y.J. (2017). RNAi-Induced Electrophysiological and Behavioral Changes Reveal two Pheromone Binding Proteins of *Helicoverpa armigera* Involved in the Perception of the Main Sex Pheromone Component Z11-16:Ald. J. Chem. Ecol..

[42] Guo M., Du L., Chen Q., Feng Y., Zhang J., Zhang X., Tian K., Cao S., Huang T., Jacquin-Joly E. (2021). Odorant receptors for detecting flowering plant cues are functionally conserved across moths and butterflies. Mol. Biol. Evol..

[43] Cheng L., Luo J., Li P., Yu H., Huang J., Luo L. (2014). Microbial diversity and flavor formation in onion fermentation. Food Funct..

[44] He H.J., Wang X.L., Zhang J.L. (2004). Analysis of volatile components of shallots by GC-MS. J. Anal. Test..

[45] Li M.F., Li T., Li W., Yang L.D. (2015). Changes in antioxidant capacity, levels of soluble sugar, total polyphenol, organosulfur compound and constituents in garlic clove during storage. Ind. Crops Prod..

[46] Chen L.H., Tian K., Wang G.R., Xu X.L., He K.H., Liu W., Wu J.X. (2020). The general odorant receptor GmolOR9 from *Grapholita molesta* (Lepidoptera: Tortricidae) is mainly tuned to eight host-plant volatiles. Insect Sci..

[47] Jiang N.J., Tang R., Wu H., Xu M., Ning C., Huang L.Q., Wang C.Z. (2019). Dissecting sex pheromone communication of *Mythimna separata* (Walker) in North China from receptor molecules and antennal lobes to behavior. Insect Biochem. Mol. Biol..

[48] Wang X., Wang S., Yi J., Li Y., Liu J., Wang J., Xi J. (2020). Three host plant volatiles, hexanal, lauric acid, and tetradecane, are detected by an antenna-biased expressed odorant receptor 27 in the dark black chafer *Holotrichia parallela*. J. Agric. Food Chem..

[49] Fan J., Zhang Y., Francis F., Cheng D., Sun J., Chen J. (2015). Orco mediates olfactory behaviors and winged morph differentiation induced by alarm pheromone in the grain aphid, *Sitobion avenae*. Insect Biochem. Mol. Biol..

[50] Hallem E.A., Ho M.G., Carlson J.R. (2004). The molecular basis of odor coding in the *Drosophila* antenna. Cell.

[51] Wang G., Carey A.F., Carlson J.R., Zwiebel L.J. (2010). Molecular basis of odor coding in the malaria vector mosquito *Anopheles gambiae*. Proc. Natl. Acad. Sci. USA.

