# Correction: Zhang et al. Expression Profiling and Functional Analysis of Candidate Odorant Receptors in *Galeruca daurica*. *Insects* 2022, *13*, 563

**DOI:** 10.3390/insects16070672

**Published:** 2025-06-27

**Authors:** Jing-Hang Zhang, Ling Li, Na Li, Yan-Yan Li, Bao-Ping Pang

**Affiliations:** 1 Research Center for Grassland Entomology, Inner Mongolia Agricultural University, Hohhot 010018, China; zhangjinghang0508@163.com (J.-H.Z.); lling@imau.edu.cn (L.L.); liyanyan@imau.edu.cn (Y.-Y.L.); 2 Erdos City Extension Center for Agriculture and Animal Husbandry Technology, Erdos 017200, China; lina445899244@163.com

In the original publication [1], there was a mistake in Table S2. The *dsGFP* primers were listed incorrectly. The corrected Table S2 appears below. The authors state that the scientific conclusions are unaffected. This correction was approved by the Academic Editor. The original publication has also been updated.

**Table S2:** List of primers used in dsRNA synthesis.

Name	Forward Primer (5′ to 3′)	Reverse Primer (5′ to 3′)
**ds*GdauOR4***	TAATACGACTCACTATAGGATGATGATGCATATTTCTC	TAATACGACTCACTATAGGTCACATCATTTGTGTTAATAG
**ds*GdauOR11***	TAATACGACTCACTATAGGGAGCGAAGAAATACGAAGTGCCT	TAATACGACTCACTATAGGGATGTTGTTCCCTGAACGCTTCT
**ds*GdauOR15***	TAATACGACTCACTATAGGGGTTGTAAGTGCCCAACTGTATA	TAATACGACTCACTATAGGGCACGTTGTGAAGAGTTCAGTTA
**ds*GdauORco***	TAATACGACTCACTATAGGATTAAATACTGGGTCGAAAGAC	TAATACGACTCACTATAGGGAAATAGGTAACTACAGCACC
**ds*GFP***	TAATACGACTCACTATAGGGCACAAGTTCAGCGTGTCCG	TAATACGACTCACTATAGGGTTCACCTTGATGCCGTTC

The underlined parts are the T7 promoter sequences.
