# Supplementary material for: Expression Profiling and Functional Analysis of Candidate Odorant Receptors in Galeruca daurica
Source: Insects. 2022 Jun 21;13(7):563. doi: 10.3390/insects13070563 (PMC9318772; doi:10.3390/insects13070563)
Supplement: Supplementary file 1 [file insects-13-00563-s001.zip › insects-1735325-supplementary-update.pdf]

Table S1. List of primers used for qRT-PCR

Gene Name	Forward Primer (5' to 3')	Reverse Primer (5' to 3')
OR1	CGCGGCTTTAGATACAGAGG	TCCAACGGGTTTAAAGTTGC
OR2	TTTAAACCCGCTCGAACTTG	TCGCTGTGAAAAATGCAGTC
OR3	TGTTGATTGTGGCGTAGGAA	TGAAAAGGGAGAATGCCACT
OR4	CTAACGGGACCGTTCATAGG	ACCAATTGATGCGTTGCATA
OR5	TGCCGTACAGGTTATCACGA	ACAGCAATTCCCTGTTTTCG
OR6	ATATGAGTGCCCTTGGATGG	TTAGGGCTACCGCCACTAGA
OR7	GTCCTGCATGCAACTGTACG	AATTATGGGATGGCTCCATTT
OR8	TTCCAAGCTAACTGGCACAA	TTGAGAACATTAATTTGAGCACCT
OR9	ATCAGCGAGCGTCTATGGTC	CAAATTTGGCTCCCTTTGTT
OR10	TTTACCATGTTCGCCATGTG	CGCCCCAATTTCAATAACAG
OR11	ACATCAGCGAGCGAAGAAAT	GTGCTCAACGTCAGTGGAGA
OR12	AATACGAAGGGCATTGTTCG	AAATGATCCCCGGCAAAC
OR13	TTCCAGAGTCAAGAGATCACTCA	AAATGTTTCCAGTGTTAAGTCGAA
OR14	TTCGATTTACGAGAGCGAATG	GCTTTCATCGATGTGACTGC
OR15	TGGTTGTAAGTGCCCAACTG	TGCACGTTGTGAAGAGTTCAG
OR16	CAAGATGCGCCAAATCATCT	TTCTGCCTTGAAACTGCTCA
OR17	AATGTCCGTGGCTAAATTGG	CAACCGCCGAGAAACTGTAT
OR18	CACACAGTTGTTCTTATTTTCATGG	TGCAGGTAAATGGCTTTTGA
OR19	TGGTGTTACTACGGCAATGAA	TCCCCGCTTGAATTTTTGTA
OR20	GAAAATCCATGGCAAATTTCA	AGGCGACAGCTGCAAGACTA
ORco	TGCATACAGTTGCCATTGGT	AGTTGCACCAACACCATGAA
SDHA	GGGAGACCACAATCTCCTCA	AGCTGGTGCTCCTAAGTCCA

Table S2. List of primers used in dsRNA synthesis.

Name	Forward Primer (5′ to 3′)	Reverse Primer (5′ to 3′)
**ds*GdauOR4***	TAATACGACTCACTATAGGAT<br>GATGATGCATATTTCTC	TAATACGACTCACTATAGGTCACAT<br>CATTTGTGTTAATAG
**ds*GdauOR11***	TAATACGACTCACTATAGGGA<br>GCGAAGAAATACGAAGTGCCT	TAATACGACTCACTATAGGGATGTT<br>GTTCCCTGAACGCTTCT
**ds*GdauOR15***	TAATACGACTCACTATAGGGG<br>TTGTAAGTGCCCAACTGTATA	TAATACGACTCACTATAGGGCACGT<br>TGTGAAGAGTTCAGTTA
**ds*GdauORco***	TAATACGACTCACTATAGGAT<br>TAAATACTGGGTCGAAAGAC	TAATACGACTCACTATAGGGAAATA<br>GGTAACTACAGCACC
**ds*GFP***	TAATACGACTCACTATAGGGC<br>ACAAGTTCAGCGTGTCCG	TAATACGACTCACTATAGGGTTCAC<br>CTTGATGCCGTTC

The underlined parts are the T7 promoter sequences.
